# IT COULD BE ANYTHING YOU WANT!: VITAL INVOLVEMENT OF PERSONS WITH DEMENTIA IN CREATIVE GROUP STORYTELLING

**DOI:** 10.1093/geroni/igac059.1486

**Published:** 2022-12-20

**Authors:** Kyong Hee Chee, Seoyoun Kim, Olga Gerhart

**Affiliations:** Texas State University, San Marcos, Texas, United States; Texas State University, San Marcos, Texas, United States; Texas State University, San Marcos, Texas, United States

## Abstract

The benefit of vital involvement (VI) in dementia care is largely unknown. The purpose of this study is to demonstrate VI elements in a creative group storytelling program for persons living with dementia (TimeSlips). After offering 6 weekly storytelling sessions for 4 small groups in a memory care community, we interviewed participants (n = 21), family members (n = 2), and care associates (n = 6) to obtain feedback on the program. Themes from narratives in the audiorecorded and transcribed interviews suggest that participants enjoyed the program, enacting their personal values, strengths, and interests, and incorporating their past and current experiences, as supported by their sociospatial environment (e.g., facilitator, co-participants, researchers, shared table). When paired with the VI practice, creative group storytelling has potential to magnify favorable outcomes for participants with dementia, who may express more fully their meaningful engagement with their inner (psychological) and external (social and physical) world.

